# The Landscape of Actionable Genomic Alterations by Next-Generation Sequencing in Tumor Tissue Versus Circulating Tumor DNA in Chinese Patients With Non-Small Cell Lung Cancer

**DOI:** 10.3389/fonc.2021.751106

**Published:** 2022-02-22

**Authors:** Jun Cai, Huihui Jiang, Shuqing Li, Xiaoxia Yan, Meng Wang, Na Li, Cuimin Zhu, Hui Dong, Dongjuan Wang, Yue Xu, Hui Xie, Shouxin Wu, Jingwei Lou, Jiangman Zhao, Qingshan Li

**Affiliations:** ^1^ Department of Oncology, First Affiliated Hospital of Yangtze University, Jingzhou, China; ^2^ Zhangjiang Center for Translational Medicine, Shanghai Biotecan Pharmaceuticals Co., Ltd., Shanghai, China; ^3^ Department of General Surgery, Yucheng Hospital of Traditional Chinese Medicine, Dezhou City, China; ^4^ Department of Oncology, Affiliated Hospital of Chengde Medical University, Chengde, China

**Keywords:** NSCLC, tissue, ctDNA, genomic subtyping, targeted therapy response

## Abstract

**Background:**

Circulating tumor DNA (ctDNA) sequence analysis shows great potential in the management of non-small cell lung cancer (NSCLC) and the prediction of drug sensitivity or resistance in many cancers. Here, we drew and compared the somatic mutational profile using ctDNA and tumor tissue sequence analysis in lung adenocarcinoma (LUAD) and squamous cell carcinoma (LUSC), and assess its potential clinical value.

**Methods:**

In this study, 221 tumor tissues and 174 plasma samples from NSCLC patients were analyzed by hybridization capture-based next-generation sequencing (NGS) panel including 95 cancer-associated genes. Tumor response assessments were applied to 137 patients with advanced-stage (III and IV) NSCLC who first received targeted agents.

**Results:**

Twenty significantly mutated genes were identified such as *TP53, EGFR, RB1, KRAS, PIK3CA, CD3EAP, CTNNB1, ERBB2, APC, BRAF, TERT, FBXW7*, and *HRAS*. Among them, *TP53* was the most frequently mutated gene and had a higher mutation probability in male (p = 0.00124) and smoking (p < 0.0001) patients. A total of 48.35% (191/395) of NSCLC patients possessed at least one actionable alteration according to the OncoKB database. Although the sensitivity of genomic profiling from ctDNA was lower than that from tumor tissue DNA, the mutational landscape of target genes from ctDNA is similar to that from tumor tissue DNA, which led to 61.22% (30/49) of mutational concordance in NSCLC. Additionally, the mutational concordance between tissue DNA and ctDNA in LUAD differs from that in LUSC, which is 63.83% versus 46.67%, indicating that NSCLC subtypes influence the specificity of mutation detection in plasma-derived ctDNA. Lastly, patients with *EGFR* and *TP53* co-alterations showed similar responses to Gefitinib and Icotinib, and the co-occurring *TP53* mutation was most likely to be a poor prognostic factor for patients receiving Gefitinib, indicating that the distributions and types of *TP53* mutations may contribute to the efficacy and prognosis of molecular targeted therapy.

**Conclusions:**

As a promising alternative for tumor genomic profiling, ctDNA analysis is more credible in LUAD than in LUSC. Genomic subtyping has strong potential in prognostication and therapeutic decision-making for NSCLC patients, which indicated the necessity for the utility of target NGS in guiding clinical management.

## Introduction

Genomic analysis is gaining popularity for targeted therapy in a wide variety of tumors (1). The biomarkers for targeted therapy can be identified by sequencing the tumor-derived DNA (2–4). However, although tissue sequencing is still the gold standard for genomic profiling, tumor tissues are not always sufficient for sequencing after histological diagnosis in advanced or metastatic patients mainly because of the inaccessibility of some tumor sites and/or the unsafety of invasive biopsy procedures (5). Furthermore, the single-region tissue sampling may not uncover the complete molecular profiling, since heterogeneous tumor subclones exist in many cancers (6–8).

DNA from both primary and metastatic tumor cells is released into the blood during the process of necrosis, apoptosis, and lysis (9). Thus, circulating tumor DNA (ctDNA) sequence analysis, a non-invasive liquid biopsy approach (5), has shown enormous potential in genomic profiling, such as identifying targetable alternations (10–12), disease surveillance (13, 14), and the monitor of minimal residual disease (15, 16). Particularly, ctDNA has been regarded as a potential biomarker analysis in non ***-*** small cell lung cancer (NSCLC) patients, whose plasma cell-free DNA (cfDNA) concentration is higher than that in healthy individuals (17–19).

As a common and highly heterogeneous malignant tumor, NSCLC accounts for about 85% of all cases of lung cancer (20, 21). It remains the leading cause of cancer death worldwide, and its 5-year overall survival rate for advanced or metastatic patients was <5% (22). Fortunately, disease progression has been significantly blocked, and the 5-year overall survival rate has also been notably improved on account of the utilization of actionable genomic alterations in advanced-stage NSCLC (23). For example, targeted therapeutics have contributed to the higher survival rates compared with standard conventional chemotherapy in metastatic NSCLC (1, 23–25). Next-generation sequencing (NGS) is regarded as a comprehensive approach to identifying a large number of uncommon but actionable mutations (26), which is increasingly recommended by many national and international clinical guidelines (27). So far, the molecular biomarkers recommended by clinical guidelines mainly include *EGFR, ERBB2 (HER2)*, and *KRAS* activating mutations; *ALK* and *ROS1* fusions; *BRAF V600E* and *MET* copy number gain (CNG) and exon 14 skipping (E14skip) mutations; and *RET* and *NTRK* rearrangements (28–30). However, about 20%–30% of NSCLC patients do not show any objective responses to epidermal growth factor receptor-tyrosine kinase inhibitors (EGFR-TKIs). Moreover, despite initial significant responses, the genetically driven tumor will eventually be resistant to targeted therapy, which severely limits the effectiveness of TKIs (31).

Co-occurring genetic alterations might contribute to explaining the molecular mechanisms of tumor resistance (32, 33). In several studies, a potential role of *TP53* mutation is associated with poor therapeutic responses (34–36). As the “guardian of the genome” (37), p53 is a DNA binding protein, which restrains the proliferation of cells with damaged DNA (38, 39). In addition, p53 is also a tumor-suppressor protein, which is regarded as the “coordinator of the underlying processes of the hallmarks of cancer”, since its inactivation paves the path for malignancy (40). However, the impact of *TP53* mutations on clinical outcomes in NSCLC patients with *EGFR*-mutation treated with first-line TKIs requires further elucidation (36), and the molecular mechanism among them remains largely unknown. In this study, we explored whether ctDNA analysis could serve as a credible alternative for tumor genomic profiling in lung adenocarcinoma (LUAD) or lung squamous cell carcinoma (LUSC), and whether the poor therapeutic response to EGFR-TKIs is associated with types and distributions of *TP53* mutations.

## Materials and Methods

### Patients and Samples Collection

We recruited 395 NSCLC patients from the Department of Oncology in Affiliated Hospital of Chengde Medical University (Chengde, China) and the Department of Oncology in First Affiliated Hospital of Yangtze University (Jingzhou, China) between February 2017 and November 2019. The pathological diagnosis was verified by independent pulmonary pathologists based on the 4th edition of the World Health Organization Classification of Lung Tumors (41), and tumors with histological components other than NSCLC were excluded. Eight fresh tumor tissues, 213 formalin-fixed and paraffin-embedded (FFPE) tumor specimens, and 174 plasma were collected for NGS analysis. For the group of targeted therapy in patients with advanced-stage (IIIB and IV) NSCLC, 69 tumor tissues and 68 plasma samples were collected before the initiation of any therapies. Clinical characteristics of 395 NSCLC patients are shown in **Table 1**.

**Table 1:** Clinical characteristics of patients enrolled in this study according to *TP53* status.

Clinical characteristics	No. of patients	*TP53*			*p*-value
		Wild type		Mutated	
Total	395	218	177		
Sample					
Tissue	221	101	120		0.00003022****
ctDNA	174	117	57		
Gender					
Male	227	109	118		0.00124***
Female	168	109	59		
Age median (range)	62 (36–85)	62 (36–85)	63 (36–85)		
Stage					
I	27	19	8		0.1302
II	14	5	9		
III	47	22	25		
IV	255	133	122		
Smoking					
Yes	162	73	89		0.000000004034***
No	224	170	54		
Former	6	3	3		
Survival					
Yes	307	163	144		0.689
No	47	27	20		
Metastasis					
Yes	273	146		127	0.5565
No	90	52		38	
Stage III or IV diseases; receiving different targeted agents (n = 139)					
Erlotinib	5	1		4	0.04139
Gefitinib	57	29		28	
Icotinib	47	23		24	
Afatinib	5	0		5	
Anlotinib	11	1		10	
Bevacizumab	6	1		5	
Camrelizumab	1	0		1	
Crizotinib	7	3		4	

Patients whose clinical data were missing were not shown.

***p < 0.001, ****p < 0.0001.

### DNA Extraction and Quality Control

Genomic DNA (gDNA) from the fresh tumor tissues, FFPE tumor specimens, and plasma was extracted by QIAamp DNA Mini Kit (Qiagen, Hilden, Germany), GeneRead DNA FFPE Kit (Qiagen, Hilden, Germany), and HiPure Circulating DNA Midi Kit C (Magen, Guangzhou, China), respectively. Qubit^®^ 3.0 Fluorometer (Invitrogen, Carlsbad, CA, USA) and NanoDrop ND-1000 (Thermo Scientific, Wilmington, DE, USA) were used to assess the quantity and purity of gDNA. Fragmentation status was evaluated by the Agilent 2100 Bioanalyzer instrument (Agilent Technologies) using the High-Sensitivity DNA Reagent (Agilent Technologies, Santa Clara, CA, USA) to produce a DNA integrity number (DIN). Additionally, the step of quality control (QC) was also performed to assess FFPE DNA integrity by multiplex polymerase chain reaction (PCR). In brief, gDNA (30 ng) was amplified using three different primers of the glyceraldehyde-3-phosphate dehydrogenase (GAPDH) gene, the size set of which was 200–400 base pairs. An Agilent 2100 bioanalyzer instrument (Agilent Technologies) was used to determine the concentration of multiplex PCR products. The fragmentation of gDNA from FFPE was estimated by the average yield ratio (AYR) value, which was calculated by dividing the yield ratio of reference DNA (Promega Madison, WI, USA) into each amplicon.

### Library Preparation and Hybridization Capture

Three hundred nanograms of gDNA from each sample determined by Qubit quantification was mechanically fragmented via an E220 focused ultrasonicator Covaris (Covaris, Woburn, MA, USA). The targeted size of the DNA fragment was between 150 and 200 bp. Then, 10–100 ng DNA was used for library construction by the KAPA library preparation kit (Kapa Biosystems Inc., Wilmington, MA, USA), which was constructed with end repair, A-tailing, and adapter ligation without additional fragmentation following the manufacturer’s instructions. Finally, the NGS libraries were captured using the xGen Lockdown Probe pool (IDT Technologies), and the captured DNA fragments were amplified with 12–13 cycles of PCR using 1× KAPA HiFi Hot Start Ready Mix.

### Illumina Sequencing

After QC and quantification by Agilent 2100 Bioanalyzer (Agilent Technologies) and Qubit^®^ 3.0 Fluorometer (Invitrogen; Thermo Fisher Scientific, Inc.), the NGS libraries were sequenced on an Illumina NextSeq CN500 platform (Illumina Inc, San Diego, CA, USA) Medium flux chip.

### Bioinformatics Analysis

Clean data were obtained following filtering adapter, low quality reads, and reads with length <36 bp, and they were aligned to reference human genome (University of California Santa Cruz ID: hg19) using the Burrows–Wheeler Aligner v. 0.7.12. Subsequently, the Picard and Genome Analysis Toolkit (GATK v.3.2) method was used for duplicate removal, local realignment, and base quality score recalibration, and it also generated the quality statistics, such as mapped reads, mean mapping quality, and mean coverage. Finally, VarDict was adopted for the identification of SNV and InDel.

The ANNOVAR software tool was used for annotating somatic variants. The candidate somatic variants were identified by the following filter conditions: (i) remove mutations with coverage depth <10×; (ii) remove variant sites with mutant allele frequency (MAF) >0.001 in the 1,000 Genomes databases (1,000 Genomes Project Consortium; https://www.internationalgenome.org/) and remove variant sites with MAF >0.001 in the ExAC (https://ncbiinsights.ncbi.nlm.nih.gov/tag/exac/); (iii) retain variant sites with MAF ≥0.001 and <0.1 in the 1,000 Genomes databases with COSMIC evidence; (iv) retain variations in the exonic or splicing region (10 bp upstream and downstream of splicing sites); (v) remove synonymous mutations; (vi) remove Unknown Variant_Classification; and (vii) the functional benign variant sites predicted by PolyPhen-2 and MutationTaster were removed.

CNVkit was employed to identify copy number variations (CNVs). Focal copy number events were determined with Genomic Identification of Significant Targets in Cancer (GISTIC) 2.0 (threshold: q-value <0.25). The correlation between the identified somatic variants and their clinical significance was established by OncoKB Precision Oncology Database (http://oncokb.org/).

### Statistical Analysis

The maftools package in R software (R 3.5.1, R Core Team; https://www.RProject.org) was used to create the mutational landscape, including somatic single nucleotide variations (SNVs) and InDels. The enrichment analysis of Kyoto Encyclopedia of Genes and Genomes (KEGG) and Gene Ontology (GO) were carried out to explore the biological significance of the candidate mutant genes by ClusterProfiler package (http://bioconductor.org/packages/release/bioc/html/clusterProfiler.Html) (42).

Tumor responses (CR, complete remission; PR, partial response; SD, stable disease; and PD, progressive disease) were assessed by the Response Evaluation Criteria in Solid Tumors (RECIST version 1.1) (43–46). The disease control rate (DCR) was defined as all positive responders (including complete, partial, and stable responders) divided by the total number of response-evaluable patients. The response rate (RR) was calculated by dividing complete and partial responders into the total number of response-evaluable patients. Meanwhile, the clinic pathological characteristics and DCR of targeted therapy were compared between two groups via chi-square tests by R software and GraphPad Prism (v. 7.0; GraphPad Software, La Jolla, CA) software, respectively. Fisher exact test was used to evaluate the statistical differences in categorical variables between two groups. A two-sided p-value <0.05 was used to evaluate statistical significance. The group of fewer than five samples was not adopted to perform statistical analyses. Tumor response assessments were only applied to patients with advanced-stage (IIIB and IV) NSCLC and receiving the first-line targeted therapy.

## Results

### Patient Characteristics

In this retrospective study, 395 NSCLC patients were enrolled including 127 patients from the Department of Oncology in Affiliated Hospital of Chengde Medical University and 268 patients from the Department of Oncology in First Affiliated Hospital of Yangtze University. Among them, 340 patients were diagnosed as LUAD, 54 patients were diagnosed as LUSC, and 1 patient was diagnosed as large cell carcinoma. In terms of tumor staging, the number of NSCLC patients in stages I–IV was 27, 14, 47, and 255, respectively. One hundred thirty-seven patients with advanced-stage (III and IV) NSCLC were treated with targeted therapy before any other therapies. For other clinical indicators, 273 of 395 cases had lymph node metastasis pathologically, 224 of 395 patients had never smoked, and 227 were male patients. Till the last time of our follow-up, 307 patients were still alive, 47 patients had died, and others were lost to follow-up (**Table 1**).

### Landscape of Somatic Mutations

**Figure 1:**
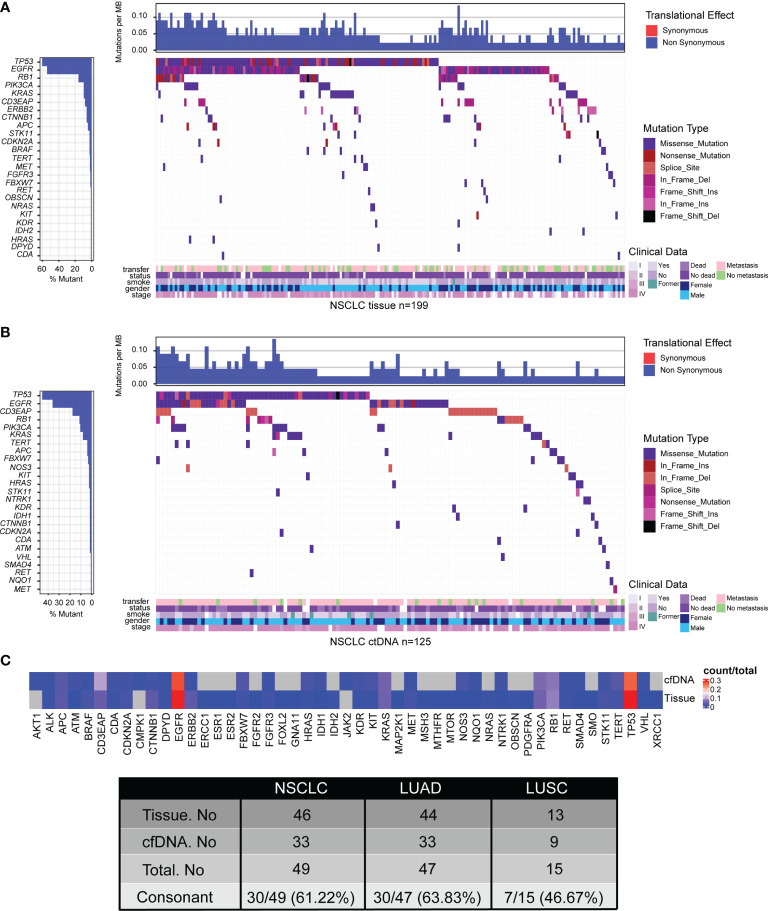
Tumor mutation landscape of NSCLC derived from tumor tissue DNA (n = 213) **(A)** and ctDNA (n = 139) **(B)**. Concordance of mutated genes derived from tumor tissue DNA or ctDNA in NSCLC patients **(C)**. Patients were arranged along the x-axis. Significantly mutated genes identified by VarDict were ranked by mutation frequencies and clinical characteristics. Tumor mutation burden (TMB, mutations per Mb) is shown in the upper panel. The bars on the left represent the mutation frequency of each gene.

To delineate the mutation landscape of NSCLC, we first analyzed somatic mutations from 221 tumor tissue samples by an NGS panel of 95 known cancer genes (**Supplementary Table S1**). A total of 455 somatic variants in 46 genes were detected in 199 of 221 (90.05%) tumor tissues, including 39 nonsense mutations, 298 missense mutations, 7 frameshift insertions, 3 frameshift deletions, 17 in-frame insertions, 79 in-frame deletions, and 12 splice sites. The top 10 frequent mutated genes were *TP53* (54.30%), *EGFR* (48.87%), *RB1* (14.48%), *KRAS* (8.60%), *PIK3CA* (8.60%), *CD3EAP* (7.24%), *CTNNB1* (5.43%), *ERBB2* (5.43%), *APC* (4.07%), and *BRAF* (2.26%) (**Figure 1A**; **Supplementary Table S2**).

To explore the feasibility of genomic profiling by peripheral blood, the NGS panel was also applied to the analysis of 174 plasma samples. A total of 236 somatic variants in 33 genes were identified in 125 of 174 (71.84%) plasma-derived ctDNA samples, including 11 nonsense mutations, 157 missense mutations, 4 frameshift insertions, 1 frameshift deletion, 3 in-frame insertions, 51 in-frame deletions, and 9 splice sites, which was lower than that from tumor tissue DNA (**Figure 1B**; **Supplementary Table S2**). Expectedly, the mutation frequencies of the most 10 common mutated genes were also lower, which were *TP53* (32.76%), *EGFR* (25.86%), *CD3EAP* (12.64%), *RB1* (8.05%), *PIK3CA* (7.47%), *KRAS* (5.75%), *APC* (2.87%), *TERT* (2.87%), *FBXW7* (2.30%), and *HRAS* (1.72%) (**Figure 1B**; **Supplementary Table S2**). In short, the percentage of ctDNA-specific mutated genes and tissue DNA-specific mutated genes were 6.12% (3/49) and 32.65% (16/49), respectively, while 61.22% (30/49) of mutated genes were shared by ctDNA analysis and tumor tissue DNA analysis (**Figure 1C**; **Supplementary Figure S1**, **Supplementary Table S2**).

### Somatic Copy Number Alterations Detection

**Figure 2:**
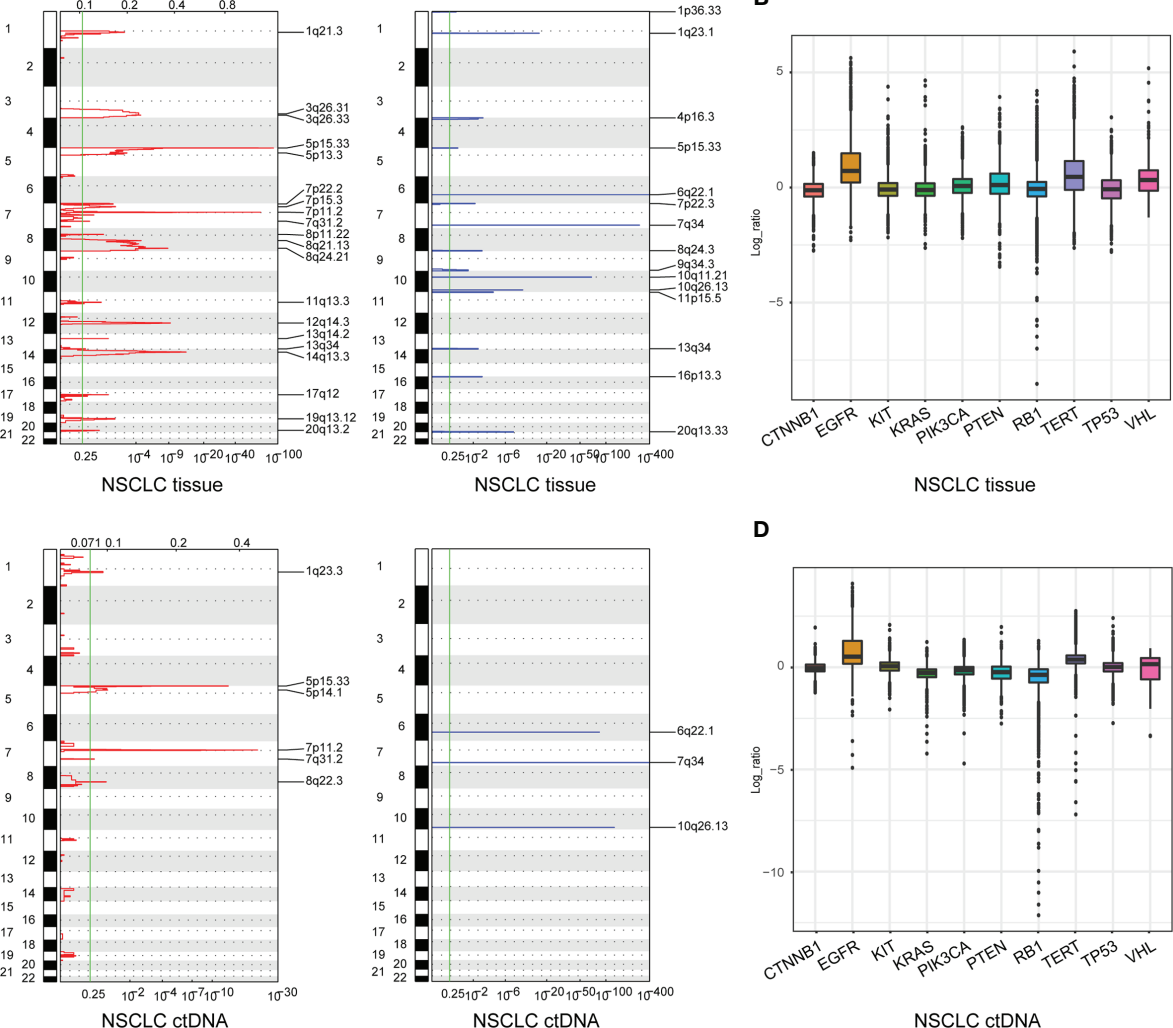
Somatic copy number alterations (SCNAs) of NSCLC derived from tumor tissue DNA (n = 213) **(A)** and ctDNA (n = 139) **(C)**. SCNAs in NSCLC are plotted by chromosomal location (vertical axis) by CNVkit. The 10 genes in the hybridization capture-based NGS panel of 95 genes **(B, D)**.

A total of 213 tumor tissue samples and 139 plasma samples were profiled for somatic copy number alterations (SCNAs) in NSCLC. There were 20 significant peaks of copy number gain in 213 tumor tissue samples including 1q21.3, 3q26.31, 3q26.33(*PIK3CA*), 5p15.33(*TERT*), 5p13.3, 7p22.2, 7p15.3, 7p11.2(*EGFR*), 7q31.2(*TP53TG1*), 8p11.22, 8q21.13, 8q24.21, 11q13.3, 12q14.3, 13q14.2(*RB1*), 13q34, 14q13.3, 17q12, 19q13.12, and 20q13.2 (**Figures 2A**, left, **2B**; **Supplementary Table S3**), while there were only six significant peaks of copy number gain in 139 plasma samples including 1q23.3, 5p15.33(*TERT*), 5p14.1, 7p11.2(*EGFR*), 7q31.2, and 8q22.3(*TP53INP1*) (**Figures 2C**, left, **2D**; **Supplementary Table S3**). Additionally, we also identified 15 significant peaks of copy number loss including 1p36.33, 1q23.1, 4p16.3, 5p15.33(*TERT*), 6q22.1, 7p22.3, 7q34, 8q24.3, 9q34.3, 10q11.21, 10q26.13, 11p15.5, 13q34, 16p13.3, and 20q13.33 in 213 tissue samples (**Figures 2A**, right, **2B**; **Supplementary Table S3**), but only three significant peaks of copy number loss including 6q22.1, 7q34 and 10q26.13 were found in 139 plasma samples (**Figures 2C**, right, **2D**; **Supplementary Table S3**). Lastly, SCNAs of LUAD (**Supplementary Figure S2**) and LUSC (**Supplementary Figure S3**) were also profiled, which further indicated that the feasibility of genomic profiling using ctDNA analysis was lower than using tumor tissue DNA analysis.

### ctDNA Analysis Has More Potential to Be an Alternative for Genomic Profiling of LUAD Compared With Its Application in LUSC

To compare the feasibility of genomic profiling using plasma samples in NSCLC subtypes, ctDNA from 143 LUAD patients and 31 LUSC patients was sequenced by the NGS panel of 95 known cancer genes.

**Figure 3:**
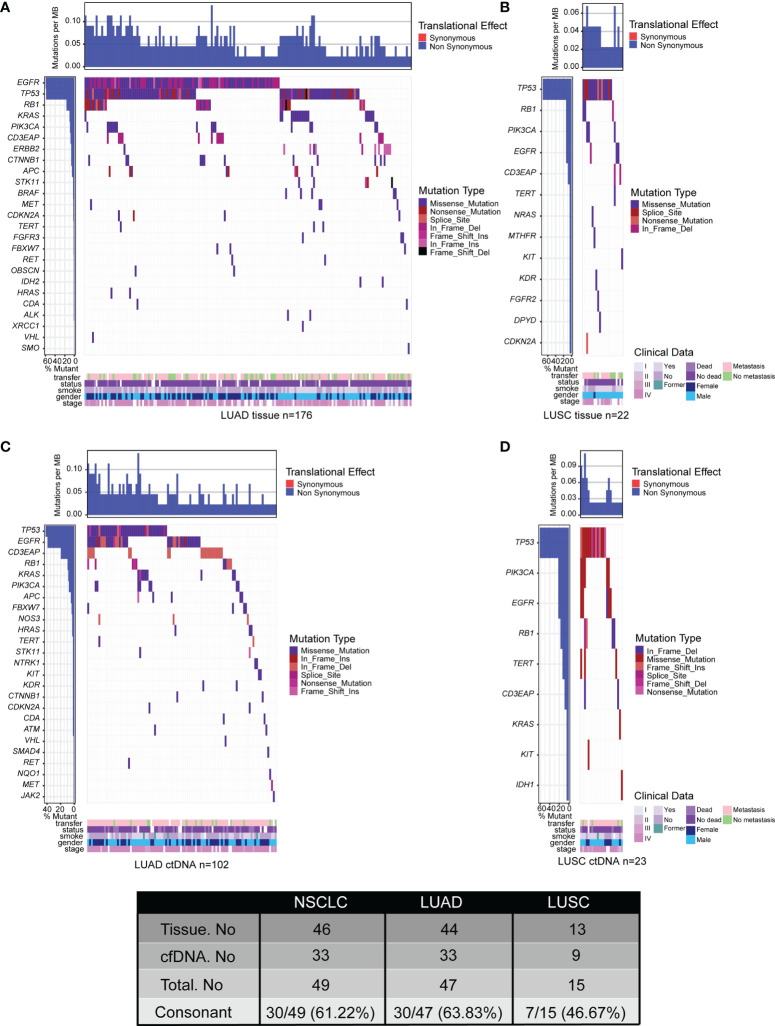
Tumor mutation landscape of NSCLC derived from LUAD tumor tissue DNA (n = 197) **(A)**, LUSC tumor tissue DNA (n = 23) **(B)**, LUAD ctDNA (n = 143) **(C)**, and LUSC ctDNA (n = 31) **(D)**. Patients were arranged along the x-axis. Significantly mutated genes identified by VarDict were ranked by mutation frequencies and clinical characteristics. Tumor mutation burden (TMB, mutations per Mb) is shown in the upper panel. The bars on the left represent the mutation frequency of each gene.

In the group of LUAD, a total of 417 somatic variants in 44 genes were identified in 176 of 197 (89.34%) tumor tissue samples, including 35 nonsense mutations, 271 missense mutations, 7 frameshift insertions, 3 frameshift deletions, 17 in-frame insertions, 75 in-frame deletions, and 9 splice sites (**Figure 3A**; **Supplementary Table S4**). There were 197 somatic variants in 33 genes identified in 102 of 143 (71.33%) ctDNA samples including 8 nonsense mutations, 131 missense mutations, 2 frameshift insertions, 3 in-frame insertions, 46 in-frame deletions, and 7 splice sites (**Figure 3C**; **Supplementary Table S4**). Moreover, the mutation frequencies of target genes detected by ctDNA analysis were lower than that by tumor tissue DNA analysis (**Figures 3A, C**; **Supplementary Table S4**). In summary, the percentage of ctDNA-specific mutated genes and tumor tissue DNA-specific mutated genes were 6.38% (3/47) and 29.79% (14/47), respectively, while 63.83% (30/47) of mutated genes were detected by both ctDNA analysis and tumor tissue DNA analysis (**Figure 3**; **Supplementary Table S4**).

In the group of LUSC, there were 36 somatic variants in 13 genes were identified in 22 of 23 (95.65%) tissue samples, including 3 nonsense mutations, 26 missense mutations, 4 in-frame deletions, and 3 splice sites (**Figure 3B**; **Supplementary Table S4**). Meanwhile, a total of 39 somatic variants in 9 genes were identified in 23 of 31 (74.19%) plasma samples including 3 nonsense mutations, 26 missense mutations, 5 in-frame deletions, 1 frameshift deletion, 2 frameshift insertions, and 2 splice sites (**Figure 3D**; **Supplementary Table S4**). Additionally, the mutation frequencies of target genes detected by ctDNA analysis were similar to that by tumor tissue DNA analysis (**Figures 3B, D**; **Supplementary Table S4**). Collectively, the percentage of ctDNA-specific mutated genes, tissue DNA-specific mutated genes, and mutated genes detected by both biopsies were 13.33% (2/15), 40% (6/15), and 46.67% (7/15), respectively (**Figure 3**; **Supplementary Table S4**). All of the results were consistent with previous studies that the genetic heterogeneity of LUSC is more complex than that in LUAD (47, 48). In short, our data indicated that ctDNA analysis is more feasible as an alternative for genomic profiling of LUAD than that of LUSC by comparing the consistency between ctDNA analysis and tumor DNA analysis.

### Enrichment of Somatic Mutations by KEGG and GO Analysis

**Figure 4:**
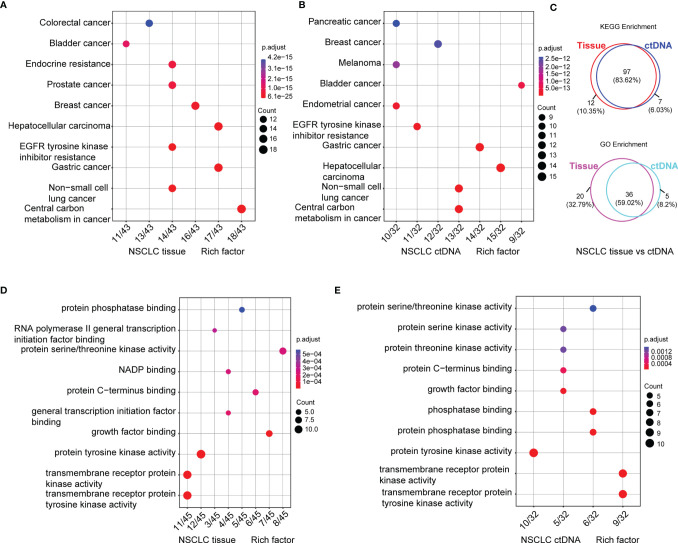
Signaling pathways by KEGG **(A, B)** and functional terms by GO **(D, E)** enrichment of somatic mutations in tumor tissue DNA (n = 213) **(A, D)** and ctDNA (n = 139) **(B, E)**. Count means the number of mutated genes enriched in this pathway. Venn diagram of signaling pathways and functional terms enriched by KEGG and GO in NSCLC, respectively (**C**, up, down).

To expound on the biological function of mutated genes in NSCLC, KEGG and GO enrichment analyses were performed. We identified 104 altered signaling pathways in 199 tumor tissues samples and 109 altered signaling pathways in 125 plasma samples by KEGG enrichment analyses (**Figures 4A, B**). All the mutated genes involved in these pathways are shown in **Table 2** and **Supplementary Table S5**. Meanwhile, a total of 56 altered functional terms were enriched in 199 tumor tissue samples, and 41 altered functional terms were enriched in 125 plasma samples by GO enrichment analyses (**Table 3**; **Supplementary Table S5**). **Figures 4D, E** show the top 10 most abundant altered functional terms according to gene counts and p-value (**Table 3**). Additionally, the mutation types and distributions of all mutated genes involved in the top 10 altered functional terms in 199 tumor tissue samples and 125 plasma samples were shown in 28 and 19 lollipop plots (**Supplementary File 2**), respectively. Collectively, we observed a high level of consistency of altered signaling pathways and altered functional terms between ctDNA analysis and tumor tissue DNA analysis, which were 83.62% (97/116) and 59.02% (36/61), respectively (**Figure 4C**).

**Table 2:** Signaling pathways enriched by KEGG analysis of somatic mutations in 199 tumor tissue samples and 125 plasma samples.

ID	Description	p.adjust	Gene
**Tumor tissue DNA**			
hsa05230	Central carbon metabolism in cancer	6.01665E−25	*EGFR/TP53/MET/PIK3CA/FGFR3/KRAS/IDH2/ERBB2/RET/HRAS/KIT/PDGFRA/NTRK1/NRAS/MAP2K1/IDH1/FGFR2/MTOR*
hsa05223	Non-small cell lung cancer	2.00456E−17	*EGFR/RB1/TP53/CDKN2A/MET/PIK3CA/KRAS/ERBB2/RET/HRAS/BRAF/NRAS/ALK/MAP2K1*
hsa05226	Gastric cancer	2.00456E−17	*EGFR/RB1/TP53/CTNNB1/MET/SMAD4/PIK3CA/KRAS/TERT/APC/ERBB2/HRAS/BRAF/NRAS/MAP2K1/FGFR2/MTOR*
hsa01521	EGFR tyrosine kinase inhibitor resistance	5.6881E−17	*EGFR/MET/PIK3CA/FGFR3/KRAS/KDR/ERBB2/HRAS/PDGFRA/BRAF/NRAS/MAP2K1/FGFR2/MTOR*
hsa05225	Hepatocellular carcinoma	9.75032E−17	*EGFR/RB1/TP53/CDKN2A/CTNNB1/MET/SMAD4/PIK3CA/KRAS/TERT/APC/HRAS/BRAF/NRAS/MAP2K1/MTOR/NQO1*
hsa05224	Breast cancer	3.02422E−16	*EGFR/RB1/TP53/CTNNB1/ESR2/PIK3CA/KRAS/APC/ERBB2/HRAS/KIT/ESR1/BRAF/NRAS/MAP2K1/MTOR*
hsa05215	Prostate cancer	6.87515E−16	*EGFR/RB1/TP53/CTNNB1/PIK3CA/KRAS/ERBB2/HRAS/PDGFRA/BRAF/NRAS/MAP2K1/FGFR2/MTOR*
hsa01522	Endocrine resistance	6.99472E−16	*EGFR/RB1/TP53/CDKN2A/ESR2/PIK3CA/KRAS/ERBB2/HRAS/ESR1/BRAF/NRAS/MAP2K1/MTOR*
hsa05219	Bladder cancer	1.27747E−15	*EGFR/RB1/TP53/CDKN2A/FGFR3/KRAS/ERBB2/HRAS/BRAF/NRAS/MAP2K1*
hsa05210	Colorectal cancer	4.15118E−15	*EGFR/TP53/CTNNB1/SMAD4/PIK3CA/KRAS/APC/MSH3/HRAS/BRAF/NRAS/MAP2K1/MTOR*
**ctDNA**			
hsa05230	Central carbon metabolism in cancer	1.76491E−17	*TP53/ERBB2/KRAS/EGFR/PIK3CA/KIT/HRAS/NTRK1/IDH1/AKT1/RET/MET/FGFR3*
hsa05223	Non-small cell lung cancer	1.76491E−17	*TP53/ERBB2/KRAS/EGFR/PIK3CA/RB1/CDKN2A/HRAS/AKT1/RET/ALK/BRAF/MET*
hsa05225	Hepatocellular carcinoma	6.99892E−16	*TP53/KRAS/EGFR/PIK3CA/RB1/APC/CDKN2A/HRAS/TERT/AKT1/CTNNB1/SMAD4/BRAF/NQO1/MET*
hsa05226	Gastric cancer	4.06853E−15	*TP53/ERBB2/KRAS/EGFR/PIK3CA/RB1/APC/HRAS/TERT/AKT1/CTNNB1/SMAD4/BRAF/MET*
hsa01521	EGFR tyrosine kinase inhibitor resistance	1.3587E−13	*ERBB2/KRAS/EGFR/PIK3CA/JAK2/HRAS/AKT1/KDR/BRAF/MET/FGFR3*
hsa05213	Endometrial cancer	2.51354E−13	*TP53/ERBB2/KRAS/EGFR/PIK3CA/APC/HRAS/AKT1/CTNNB1/BRAF*
hsa05219	Bladder cancer	5.28612E−13	*TP53/ERBB2/KRAS/EGFR/RB1/CDKN2A/HRAS/BRAF/FGFR3*
hsa05218	Melanoma	1.87101E−12	*TP53/KRAS/EGFR/PIK3CA/RB1/CDKN2A/HRAS/AKT1/BRAF/MET*
hsa05224	Breast cancer	2.50396E−12	*TP53/ERBB2/KRAS/EGFR/PIK3CA/RB1/APC/KIT/HRAS/AKT1/CTNNB1/BRAF*
hsa05212	Pancreatic cancer	2.63813E−12	*TP53/ERBB2/KRAS/EGFR/PIK3CA/RB1/CDKN2A/AKT1/SMAD4/BRAF*

**Table 3:** Functional terms enriched by GO analysis of somatic mutations in 199 tumor tissue samples and 125 plasma samples.

ID	Description	p.adjust	Gene
**Tumor tissue DNA**			
GO:0004714	Transmembrane receptor protein tyrosine kinase activity	4.33572E−16	*EGFR/MET/FGFR3/KDR/ERBB2/RET/KIT/PDGFRA/NTRK1/ALK/FGFR2*
GO:0019199	Transmembrane receptor protein kinase activity	5.25951E−15	*EGFR/MET/FGFR3/KDR/ERBB2/RET/KIT/PDGFRA/NTRK1/ALK/FGFR2*
GO:0004713	Protein tyrosine kinase activity	2.63044E−14	*EGFR/MET/FGFR3/KDR/ERBB2/RET/KIT/PDGFRA/NTRK1/ALK/MAP2K1/FGFR2*
GO:0019838	Growth factor binding	2.18377E−06	*EGFR/FGFR3/KDR/ERBB2/PDGFRA/NTRK1/FGFR2*
GO:0140296	General transcription initiation factor binding	0.000191863	*TP53/ESR1/MTOR/ERCC1*
GO:0008022	Protein C-terminus binding	0.000208465	*CTNNB1/TERT/ERBB2/HRAS/MAP2K1/ERCC1*
GO:0050661	NADP binding	0.000236227	*DPYD/IDH1/MTHFR/NOS3*
GO:0004674	Protein serine/threonine kinase activity	0.000236227	*EGFR/PIK3CA/OBSCN/STK11/BRAF/ATM/MAP2K1/MTOR*
GO:0001091	RNA polymerase II general transcription initiation factor binding	0.000316813	*TP53/ESR1/ERCC1*
GO:0019903	Protein phosphatase binding	0.000563072	*EGFR/TP53/CTNNB1/MET/ERBB2*
**ctDNA**			
GO:0004714	Transmembrane receptor protein tyrosine kinase activity	1.29478E−13	*ERBB2/EGFR/KIT/NTRK1/RET/KDR/ALK/MET/FGFR3*
GO:0019199	Transmembrane receptor protein kinase activity	8.47279E−13	*ERBB2/EGFR/KIT/NTRK1/RET/KDR/ALK/MET/FGFR3*
GO:0004713	Protein tyrosine kinase activity	1.13763E−12	*ERBB2/EGFR/KIT/JAK2/NTRK1/RET/KDR/ALK/MET/FGFR3*
GO:0019903	Protein phosphatase binding	8.65923E−06	*TP53/ERBB2/EGFR/AKT1/CTNNB1/MET*
GO:0019902	Phosphatase binding	3.30352E−05	*TP53/ERBB2/EGFR/AKT1/CTNNB1/MET*
GO:0019838	Growth factor binding	0.000112362	*ERBB2/EGFR/NTRK1/KDR/FGFR3*
GO:0008022	Protein C-terminus binding	0.000415593	*ERBB2/JAK2/HRAS/TERT/CTNNB1*
GO:0106311	Protein threonine kinase activity	0.001338361	*PIK3CA/STK11/ATM/AKT1/BRAF*
GO:0106310	Protein serine kinase activity	0.001338361	*PIK3CA/STK11/ATM/AKT1/BRAF*
GO:0004674	Protein serine/threonine kinase activity	0.001578568	*EGFR/PIK3CA/STK11/ATM/AKT1/BRAF*

**Figure 5:**
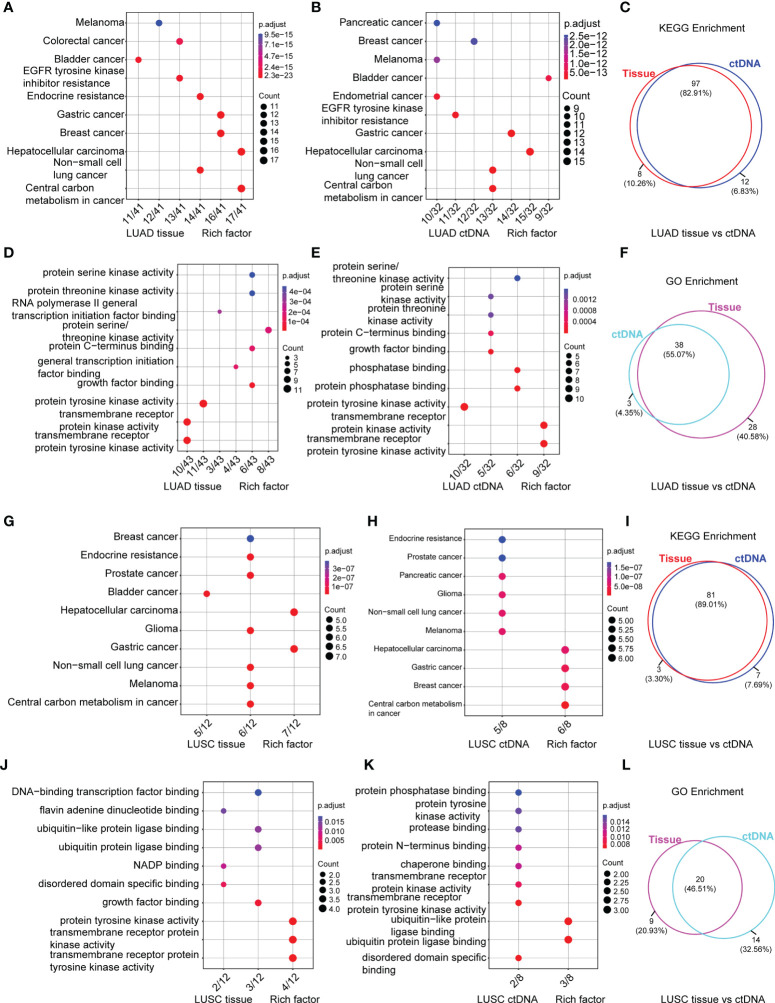
Signaling pathways by KEGG **(A, B, G, H)** and functional terms by GO **(D, E, J, K)** enrichment of somatic mutations in LUAD tumor tissue DNA (n = 197) **(A, D)**, LUAD ctDNA (n = 143) **(B, E)**, LUSC tumor tissue DNA (n = 23) **(G, J)** and LUSC ctDNA (n = 31) **(H, K)**. Count means the number of mutated genes enriched in this pathway. Venn diagram of signaling pathways **(C, I)** and functional terms **(F, L)** in LUAD **(C, F)** and LUSC **(I, L)** enriched by KEGG and GO, respectively.

Additionally, **Figure 5** shows the top 10 altered signaling pathways and altered functional terms in LUAD (**Figures 5A–F**; **Supplementary Table S6**) and LUSC (**Figures 5G**–**L**; **Supplementary Table S6**) enriched by KEGG enrichment analyses (**Figures 5A, B, G, H**) and GO enrichment analyses (**Figures 5D, E, J, K**) using tumor tissues (**Figures 5A, D, G, J**) and ctDNA (**Figures 5B, E, H, K**). A high level of consistency of altered signaling pathways between ctDNA analysis and tumor DNA analysis was observed in both LUAD and LUSC, which were 82.91% (97/117) versus 89.01% (81/91) (**Figures 5C, I**). However, we found about 50% consistency of altered functional terms between these two kinds of samples in both LUAD and LUSC, which were 55.07% (38/69) versus 46.51% (20/43) (**Figures 5F, L**). Among these significantly altered functional terms, we focused on the alteration in transmembrane receptor protein tyrosine kinases (**Figures 5D, E, J, K**), which play key roles in a wide range of cellular processes including growth, motility, differentiation, apoptosis, and metabolism (49, 50). As in previous studies, the abnormity of receptor tyrosine kinases (RTKs) signaling is closely related to diseases, especially malignancy (51–54). Constitutive activation, such as gain-of-function mutations, genomic amplification, chromosomal rearrangements, and/or autocrine activation, serves as excellent examples to show the oncogenic properties of RTKs (49, 55).

### Clinical Actionability for the Therapy of Targeted Agents

To assess the clinical utility of anticipative molecular profiling, all mutations were divided into different levels according to the evidence of clinical actionability in OncoKB (**Supplementary Figure S4A**). As standard therapeutic biomarkers, a cluster of gene mutations was approved by the FDA. In our cohort, 48.35% (191/395) of patients possess at least one actionable alteration. In the group of NSCLC, level_1 accounted for 79.21% (343/433), including missense mutation of *EGFR, PIK3CA, BRAF, IDH2, IDH1, ATM*, and *KRAS*, in-frame insertion of *EGFR*, and in-frame deletion of *EGFR*; level_2 accounted for 16.17% (70/433), including in-frame insertion of *ERBB2* and missense mutation of *ERBB2*, *PIK3CA, KRAS, BRAF*, and *NRAS*; level_3 accounted for 1.62% (7/433), including in-frame deletion of *EGFR* and missense mutation of *AKT1*; level_4 accounted for 3.00% (13/433), including missense mutation of *EGFR, FGFR3*, and *mTOR*, and nonsense mutation of *CDKN2A* (**Table 4**; **Supplementary Figures S4B, E, H**; **Supplementary Table S7**). However, patients with LUAD have a higher percentage of actionable alterations than patients with LUSC, which were 52.06% (177/340) versus 25.93% (14/54) (**Supplementary Figures S4C, D, F, G, I, J**; **Supplementary Table S7**), indicating that they may benefit more from targeted therapy.

**Table 4:** Distribution of actionable alterations in NSCLC (n = 191).

Highest_Drug	Highest_Level	Gene and Variant_Classification	Count
Afatinib	LEVEL_1	*EGFR* : Missense_Mutation:p.G452A; *EGFR* : Missense_Mutation:p.L594Q; *EGFR* : Missense_Mutation:p.G452C; *EGFR* : Missense_Mutation:p.S501I; *EGFR* : Missense_Mutation:p.G452S	30
Alpelisib+Fulvestrant	LEVEL_1	*PIK3CA* : Missense_Mutation:p.E545K; *PIK3CA* : Missense_Mutation:p.E542K; *PIK3CA* : Missense_Mutation:p.C420R;*PIK3CA* : Missense_Mutation:p.H1047R	17
Amivantamab,Mobocertinib	LEVEL_1	*EGFR* : In_Frame_Ins:p.A500delinsASVD; *EGFR* : In_Frame_Ins:p.P505delinsPHG; *EGFR* : In_Frame_Ins:p.P505delinsPH;*EGFR* : In_Frame_Ins:p.N504delinsNPHVC; *EGFR* : In_Frame_Ins:p.D503delinsDNGP; *EGFR* : In_Frame_Ins:p.H506delinsPNPY;*EGFR* : In_Frame_Ins:p.D503delinsDN	16
Dabrafenib,Dabrafenib+Trametinib	LEVEL_1	*BRAF* : Missense_Mutation:p.V600E	3
Enasidenib	LEVEL_1	*IDH2*: Missense_Mutation:p.R10Q	1
Erlotinib,Erlotinib+Ramucirumab	LEVEL_1	*EGFR* : In_Frame_Del:p.480_486del; *EGFR* : Missense_Mutation:p.L591R; *EGFR* : In_Frame_Del:p.479_483del; *EGFR* : In_Frame_Del:p.478_483del; *EGFR* : In_Frame_Del:p.479_484del; *EGFR* : In_Frame_Del:p.484_492del; *EGFR* : In_Frame_Del:p.479_481del; *EGFR* : In_Frame_Del:p.479_485del	228
Ivosidenib	LEVEL_1	*IDH1*: Missense_Mutation:p.R132C	3
Olaparib	LEVEL_1	*ATM* : Missense_Mutation:p.R2832H	1
Osimertinib	LEVEL_1	*EGFR* : Missense_Mutation:p.T523M	26
Sotorasib	LEVEL_1	*KRAS* : Missense_Mutation:p.G12C	18
Ado-Trastuzumab Emtansine,Trastuzumab Deruxtecan	LEVEL_2	*ERBB2* : In_Frame_Ins:p.E770delinsEAYVM;*ERBB2*:Missense_Mutation:p.S310F;*ERBB2*:In_Frame_Ins:p.V777delinsVGFP;*ERBB2* : In_Frame_Ins:p.V777delinsVGSP;*ERBB2*:In_Frame_Ins:p.G776delinsVC;*ERBB2*:In_Frame_Ins:p.A771delinsAYVMA	24
Alpelisib+Fulvestrant	LEVEL_2	*PIK3CA* : Missense_Mutation:p.M1043I; *PIK3CA* : Missense_Mutation:p.M1043V; *PIK3CA* : Missense_Mutation:p.V344G;*PIK3CA* : Missense_Mutation:p.R38C; *PIK3CA* : Missense_Mutation:p.G1049R; *PIK3CA* : Missense_Mutation:p.Q546H;*PIK3CA* : Missense_Mutation:p.C378F; *PIK3CA* : Missense_Mutation:p.C378R	8
Cobimetinib,Trametinib	LEVEL_2	*KRAS* : Missense_Mutation:p.K117N; *KRAS* : Missense_Mutation:p.A146T; *KRAS* : Missense_Mutation:p.G13D; *BRAF* : Missense_Mutation:p.N581I; *KRAS* : Missense_Mutation:p.K117R; *KRAS* : Missense_Mutation:p.G12D; *KRAS* : Missense_Mutation:p.A59G; *KRAS* : Missense_Mutation:p.G12A; *KRAS* : Missense_Mutation:p.G12V; *NRAS* : Missense_Mutation:p.Q61L; *BRAF* : Missense_Mutation:p.G596R; *BRAF* : Missense_Mutation:p.K601E; *MAP2K1*:Missense_Mutation:p.P124S; *NRAS* : Missense_Mutation:p.Q61R	38
Afatinib	LEVEL_3A	*EGFR* : In_Frame_Del:p.442_443del; *EGFR* : In_Frame_Del:p.484_492del	4
AZD5363	LEVEL_3A	*AKT1* : Missense_Mutation:p.E17K	3
Debio1347,Infigratinib	LEVEL_4	*FGFR3* : Missense_Mutation:p.G375D	1
Everolimus,Temsirolimus	LEVEL_4	*MTOR* : Missense_Mutation:p.C1483F	1
Lapatinib	LEVEL_4	*EGFR* : Missense_Mutation:p.A244V	5
Palbociclib,Ribociclib	LEVEL_4	*CDKN2A* : Nonsense_Mutation:p.E10X; *CDKN2A*:Nonsense_Mutation:p.S12X	6

### Targeted Therapy Response Is Related to Genomic Subtyping

**Figure 6:**
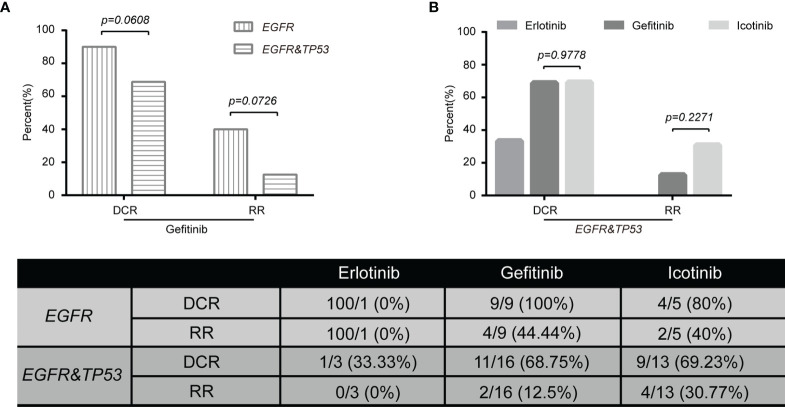
The tumor response to Gefitinib was associated with genomic subtyping in NSCLC **(A)**. The comparative analysis of p53 mutations for patients treated with targeted agents **(B)**. DCR, disease control rate (complete response + partial response + stable disease). PD, progressive disease; *EGFR*, NSCLC with *EGFR* but not *TP53* alterations (n = 15); *EGFR&TP53*, NSCLC with *EGFR* and *TP53* co-alterations (n = 32). Note: Tumor response data were only collected from patients with advanced disease (stages III and IV) who were first treated with targeted agents. The group including more than five patients was used to do statistical analyses.

Next, we wanted to explore the impact of genomic characteristics on the clinical outcomes of patients with advanced-stage (III and IV) NSCLC. Based on *EGFR* mutations, 46 patients were classified as the group of *EGFR* alterations without *TP53* mutations (called *EGFR*), and 68 patients were classified as the group of *EGFR* and *TP53* co-alterations (called *EGFR*&*TP53*). Among them, 13 out of 46 patients in *EGFR* group and 19 out of 68 patients in the *EGFR*&*TP53* group were first treated with Gefitinib. As shown in **Figure 6**, the disease control rate (DCR = CR + PR + SD) in patients with *EGFR* alterations alone and in patients with *EGFR*&*TP53* co-alterations was 100% (9/9) and 68.75% (11/16), respectively (p = 0.0608) (**Figure 6A**; **Table 5**). Meanwhile, the response rate (RR = CR + PR) was 44.44% (4/9) in patients with *EGFR* alterations alone, while only 12.5% (2/16) in patients with *EGFR*&*TP53* co-alterations (p = 0.0726) (**Figure 6A**; **Table 5**). Importantly, the distributions of drug-resistant *EGFR* mutations were also explored, and the percentage of *T790M* mutation in patients with PR, SD, and PD was 16.67% (1/6), 14.29% (2/14), and 20% (1/5), respectively (**Table 5**; **Supplementary Table S8**). Additionally, we also sought to investigate whether the *TP53* concomitant mutations are associated with the responses to different first-generation EGFR-TKIs. As shown in **Figure 6B** and **Table 5**, the DCR of Gefitinib was similar to that of Icotinib (68.75% vs 69.23%, p > 0.9778).

**Table 5:** The tumor response to targeted agents was associated with genomic subtyping in NSCLC.

Agent		*EGFR*							*TP53*	
		Sensitizing mutations		T790M status		Other mutations			Status	
		19del	L858R	Pos	Neg	c.2237_2255T	A750P	Uncommon	Pos	Neg
Erlotinib, No.	PR	0	0	0	0	0	0	1	0	0
	SD	0	0	0	0	0	0	1	1	0
	PD	2	0	1	0	0	0	0	2	0
Gefitinib, No.	PR	3	3	1	0	1	0	1	2	0
	SD	8	5	2	0	0	1	3	9	0
	PD	1	4	1	0	0	0	0	5	0
Icotinib, No.	PR	2	3	0	0	1	1	1	4	0
	SD	3	4	1	0	0	0	1	5	0
	PD	2	3	1	0	0	0	0	4	0

Tumor response data were only collected from patients with advanced disease (stage III and IV) who first received targeted agents.

PR, partial response; SD, stable disease; PD, progressive disease; Pos, patients with TP53 mutations; Neg, patients without TP53 mutations.

**Figure 7:**
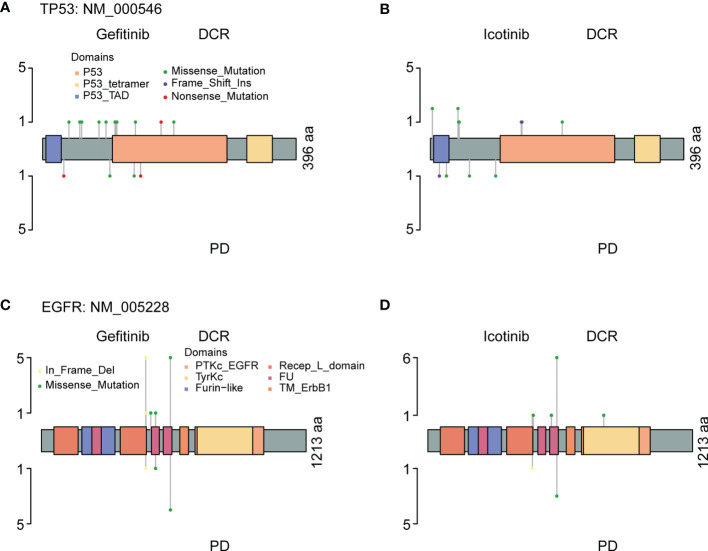
The protein structure, mutation types, and mutation distributions of TP53 **(A, B)** and EGFP **(C, D)** in the patients with *EGFR&TP53* co-alterations who were first treated with Gefitinib **(A, C)** and Icotinib **(B, D)**.

Co-occurring genetic alterations might play important roles in the mechanisms of tumor responses, and it may be helpful to explain the marked diversity of individual outcomes (32, 33). As previous studies have reported, a potential role of *TP53* mutation is associated with poor therapeutic responses (34–36). **Figure 7** shows mutations and protein structures of p53 and EGFR in NSCLC patients who were first treated with Gefitinib, Icotinib, or either of them. *TP53* mutations were detected in 11 patients who responded well to Gefitinib, including nonsense mutations (numbered BT1812060034LNCTX), and missense mutations (numbered BT1806210029LNCBP, BT1806220195LNCTV, BT1806290337LNCTB, BT1808040195LNCTV, BT1808230036LNCBP, BT1810270069LNCBP, BT1812020489LNCTS, BT1902210293LNCTV, BT1905230269LNCTV, and BT1906210049LNCBP) (**Figure 7A**, up). However, *TP53* mutation sites and types in five patients with PD were different from those in the above patients, including nonsense mutations (numbered BT1812160473LNCTV and BT1901200062LNCTB), missense mutations (numbered BT1902140231LNCTX and BT1905110010LNCBP), and splice site (numbered BT1805130336LNCTV) (**Figure 7A**, down). Furthermore, we also carried out the comparative analysis of p53 mutations for patients treated with Icotinib (**Figure 7B**; **Supplementary Table S8**) because it has similar chemical structures, molecular mechanisms, and clinical curative effects with Gefitinib but cost a much lower price. Our results also indicated that patients who responded differently to Icotinib carried different mutation sites. However, the roles of *TP53* mutations on clinical outcomes in NSCLC patients with *EGFR*-mutation treated with Gefitinib and Icotinib require further elucidation, since the molecular mechanism among them remains largely unknown.

## Discussion

Here, we identified somatic mutations in 221 tumor tissue DNA and 174 plasma-derived ctDNA by an NGS panel of 95 known cancer genes. Among them, 340, 54, and 1 out of 395 patients are LUAD, LUSC, and large cell carcinoma, respectively. The majority of these patients were at stage IV (64.56%, 255/395), where both inter- and intratumor heterogeneity had occurred. The most two common mutated genes in both tumor tissue DNA and ctDNA were *TP53* and *EGFR*. As a tumor-suppressor gene, *TP53* is frequently mutated in almost every type of cancer (40, 56, 57). *EGFR* mutation is the most frequently used marker to guide targeted therapy in NSCLC clinically (3). To sum up, four points were mainly acquired in this study. First, the feasibility of genomic profiling in NSCLC from ctDNA was lower than that from tissue DNA. Second, ctDNA analysis could serve as an alternative for genomic profiling is more credible in LUAD than that in LUSC. Third, patients with *EGFR* & *TP53* co-alterations showed similar responses to Gefitinib and Icotinib. Lastly, *TP53* concomitant mutations might be a poor prognostic factor for patients treated with Gefitinib, and the complexity of identifying *TP53* mutations may contribute to the efficacy and prognosis of molecular targeted therapy in NSCLC.

One primary purpose of this study was to explore the differences of somatic mutations detected by ctDNA analysis and tumor tissue DNA analysis in NSCLC subtypes. Comparatively speaking, the sensitivity of genetic mutation detected in plasma samples was lower than that in tumor tissue samples, 71.84% (125/174) versus 90.05% (199/221), which was consistent with previous studies (58, 59). The genomic concordance between tissue DNA and ctDNA in LUAD differs from that in LUSC, which is 63.83% versus 46.67%, indicating that NSCLC subtypes influence the specificity of mutation detection in plasma-derived ctDNA (60–62). In addition, the mutation frequencies of the 10 most common mutated genes (e.g., *EGFR*, *TP53*, *PIK3CA*, *RB1*, *CD3EAP*, *CTNNB1*, *ERBB2*, *APC*, *ERBB2*, *FGFR2*) in LUAD were different from that in LUSC, further indicating that the differences in genetic mutations exist in NSCLC subtypes (63–66). To be specific, the mutation frequency of *TP53* in LUSC was higher than that in LUAD, which was 69.56% versus 52.28% in tumor tissue DNA and 45.16% versus 30.07% in ctDNA (64). A similar trend was also observed in *PIK3CA*. On the contrary, *EGFR* mutations were more often observed in LUAD than that in LUSC, which is 53.30% versus 13.04% in tissue DNA, and 27.97% versus 16.13% in ctDNA (63). This trend was also suitable for the mutation frequencies of *KRAS* and *APC*. Collectively, the discovery of genomic pro ***fi*** ling among NSCLC subtypes (LUAD, LUSC, and large-cell carcinoma) could also benefit a lot from ctDNA.

Another primary purpose of this study was to explore the impact of *TP53* mutations on clinical outcomes in NSCLC patients with *EGFR*-mutation treated with ***fi*** rst-line TKIs, and the DCR in patients with *EGFR*&*TP53* co-alterations was lower than that in patients with *EGFR* mutations (p = 0.0608). Our results were consistent with previous studies that patients with *TP53* concomitant mutations had a lower ORR and shorter PFS of EGFR-TKI treatment compared to patients without *TP53* alterations in advanced NSCLC, and none of these studies reached statistical significance (34, 35, 67–69). Meanwhile, Qin et al. showed that concurrent *TP53* mutations could significantly reduce the responses to first-line EGFR-TKIs and were related to worse prognosis in advanced NSCLC in a meta-analysis (70). However, we were not able to confirm whether the poor prognosis is directly associated with the types or the distributions of *TP53* mutations, and this association required further elucidation, since the molecular mechanism among them remains largely unknown. Overall, growing evidence has indicated that the observed clinical heterogeneity in NSCLC patients with *EGFR* mutation may be partly due to the co-occurring molecular events. These concomitant mutations or the measurements of p53 protein levels may be used for predicting clinical outcomes in *EGFR*-mutant patients treated with targeted therapy in the future.

Liquid biopsies are a potential way to identify actionable biomarkers when they are difficult to acquire from tumor tissue samples clinically. This is mostly due to the insufficiency of available tissue, the failure of quality control, and the inadequate depth or breadth of analysis by a biomarker panel. In this present study, plasma-derived ctDNA increases detection rates of driver oncogene by 14.43%, and 57 additional patients get the opportunity to receive targeted therapy and/or participate in clinical trials. Taken together, our finding is consistent with previous studies that the identification of targetable alterations is growing in patients with the aid of plasma-derived ctDNA (71, 72). However, our study has several limitations: (i) this is a retrospective study, and its database has a certain limitation in investigating other sources of potential bias; (ii) only two institutions of accrual were included; (iii) NGS data were obtained from tumor tissues and plasma from different NSCLC patient cohorts and without matched germline DNA; (iv) survival curve analysis was not performed; (v) NSCLC patients for erlotinib response were less than 5; and (vi) the generalizability of our conclusions should be considered carefully.

## Conclusions

In conclusion, our study suggests that ctDNA analysis was regarded as an alternative for genomic profiling in advanced or metastatic patients and is more credible in LUAD than that in LUSC. NSCLC patients with *EGFR*&*TP53* co-alterations showed similar responses to Gefitinib and Icotinib, and *TP53* concomitant mutations might be a poor prognostic factor for patients with *EGFR*-mutation treated with Gefitinib. However, comprehensive molecular mechanisms about concurrent *TP53* mutations on clinical outcomes are dependent on further studies.

## Data Availability Statement

The datasets presented in this study can be found in online repositories. The names of the repository/repositories and accession number(s) can be found below: https://www.ncbi.nlm.nih.gov/sra/?term=PRJNA801361.

## Ethics Statement

The studies involving human participants were reviewed and approved by the Ethics Committee of the First Affiliated Hospital of Yangtze University, the Ethics Committee of Affiliated Hospital of Chengde Medical University. The patients/participants provided their written informed consent to participate in this study.

## Author Contributions

JC and HJ drafted the article and reviewed the submitted version of the manuscript. JC, MW, NL, CZ, HD, and DW worked on the acquisition of data. SW, JZ, HX, SL, and HJ worked on the analysis and interpretation of data. QL, JZ, SW, HJ, YX, XY and HX critically revised the article and worked on the statistical analysis. JL, JZ, and HJ are responsible for administrative/technical/material support and study supervision. QL, JZ, JL, and SW worked on conception and design. JL and QL approved the final version of the manuscript on behalf of all authors. All authors contributed to the article and approved the submitted version.

## Conflict of Interest

Authors HJ, XY, YX, HX, SW, JL and JZ were employed by Shanghai Biotecan Pharmaceuticals Co., Ltd.

The remaining authors declare that the research was conducted in the absence of any commercial or financial relationships that could be construed as a potential conflict of interest.

## Publisher’s Note

All claims expressed in this article are solely those of the authors and do not necessarily represent those of their affiliated organizations, or those of the publisher, the editors and the reviewers. Any product that may be evaluated in this article, or claim that may be made by its manufacturer, is not guaranteed or endorsed by the publisher.
