# Supplementary material for: The Landscape of Actionable Genomic Alterations by Next-Generation Sequencing in Tumor Tissue Versus Circulating Tumor DNA in Chinese Patients With Non-Small Cell Lung Cancer
Source: Front Oncol. 2022 Feb 22;11:751106. doi: 10.3389/fonc.2021.751106 (PMC8902245; doi:10.3389/fonc.2021.751106)

The protein structure, mutation types and mutation distributions of all mutated genes involved in the top 10 altered functional terms in 199 tumor tissue samples.

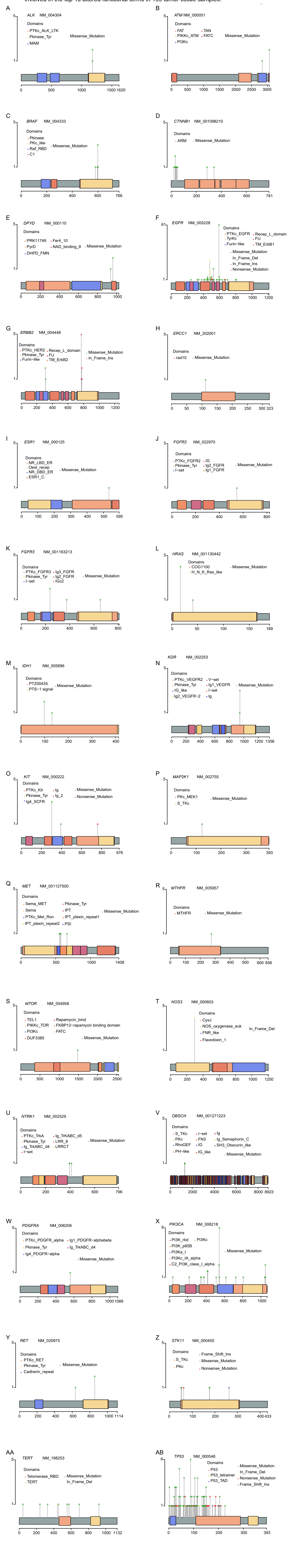

The protein structure, mutation types and mutation distributions of all mutated genes involved in the top 10 altered functional terms in 125 plasma samples.

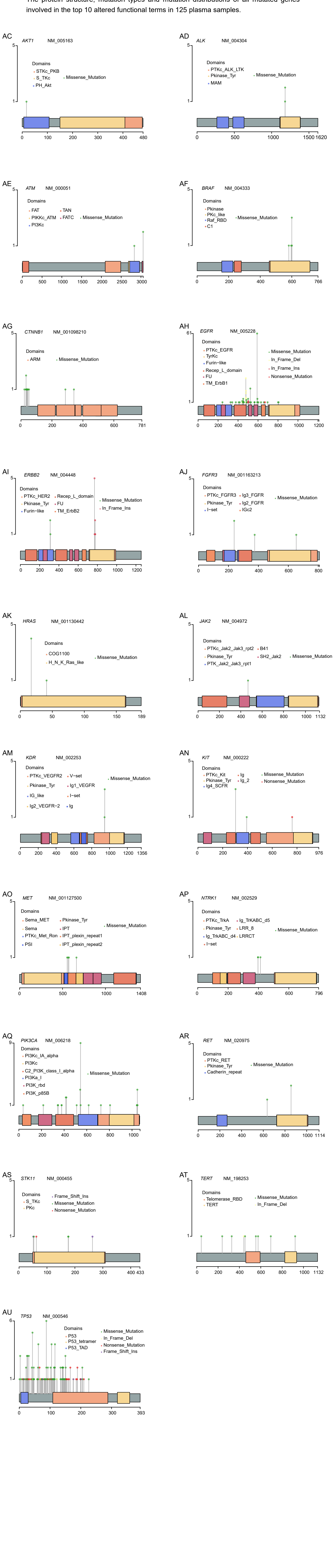

Supplement: Supplementary file 1 [file DataSheet_1.pdf]
