# Supplementary material for: The Landscape of Actionable Genomic Alterations by Next-Generation Sequencing in Tumor Tissue Versus Circulating Tumor DNA in Chinese Patients With Non-Small Cell Lung Cancer
Source: Front Oncol. 2022 Feb 22;11:751106. doi: 10.3389/fonc.2021.751106 (PMC8902245; doi:10.3389/fonc.2021.751106)
Supplement: Supplementary file 2 [file DataSheet_2.docx]

Supplementary Material

The Landscape of Actionable Genomic Alterations by Next-Generation Sequencing in Tumor Tissue versus Circulating tumor DNA in **Chinese Patients with** Non-Small Cell Lung Cancer

**Jun Cai^1,^**^†^**, Huihui Jiang^2,^**^†^**, Shuqing Li^3^, Xiaoxia** **Yan^2^,** **Meng Wang^1^****, Na Li^1^****, Cuimin Zhu****^4^, Hui Dong****^4^, Dongjuan Wang^4^, Yue Xu^2^, Hui Xie^2^, Shouxin Wu^2,*^,** **Jingwei Lou^2,*^,** **Jiangman Zhao^2,*^, Qingshan Li^4,*^**

^1^Department of Oncology，First Affiliated Hospital of Yangtze University, Jingzhou, Hubei Province, China

^2^Zhangjiang Center for Translational Medicine, Shanghai Biotecan Pharmaceuticals Co., Ltd., 180 Zhangheng Road, Shanghai, China

^3^Department of general surgery, Yucheng Hospital of Traditional Chinese Medicine, 601 Kaituo Road, Yucheng City, Dezhou City, Shandong Province, China

^4^Department of oncology, Affiliated Hospital of Chengde Medical University, Nan Ying Zi street, Chengde, Hebei province, China.

***Correspondence:**Qingshan Li

libing200865@126.com

Jiangman Zhao

zhaojiangman86@163.com

Jingwei Lou

jingweilou@biotecan.com

Shouxin Wu

swu@biotecan.com

^†^These authors have contributed equally to this work


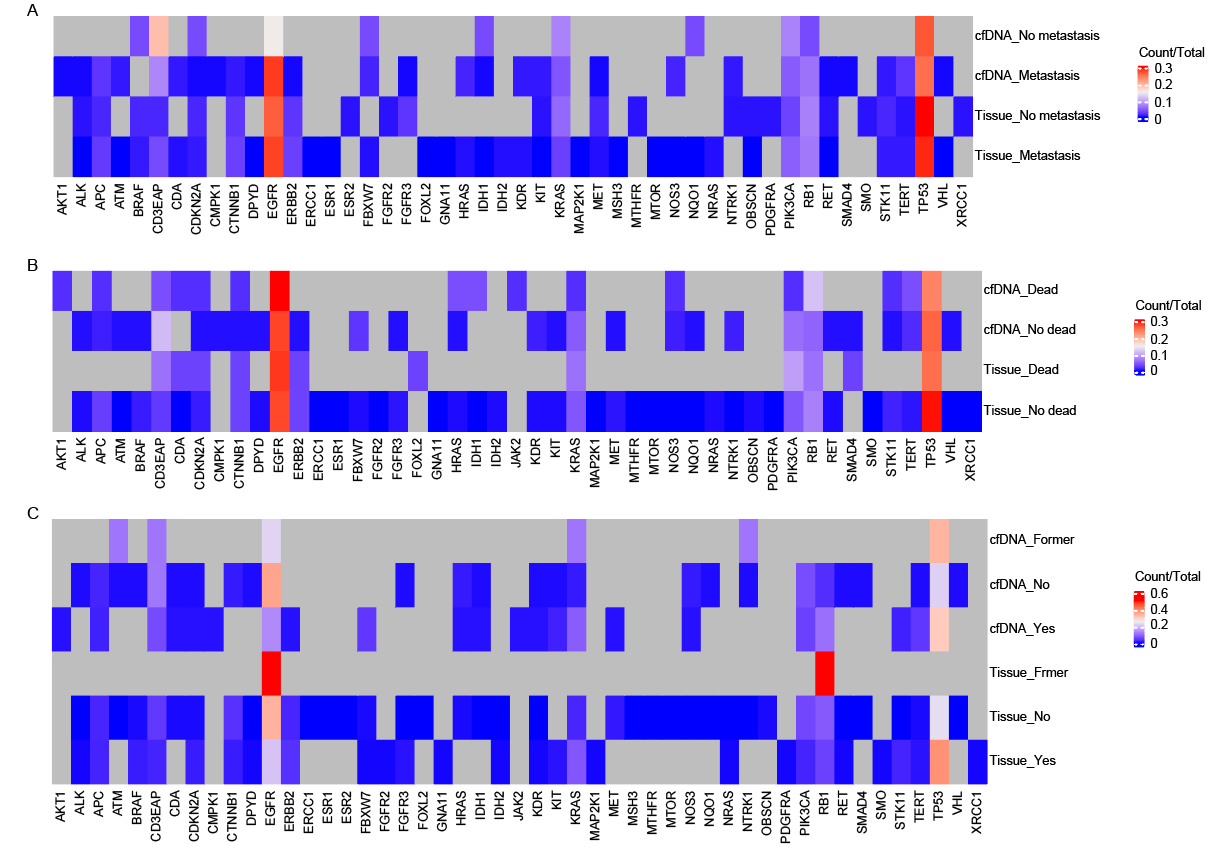

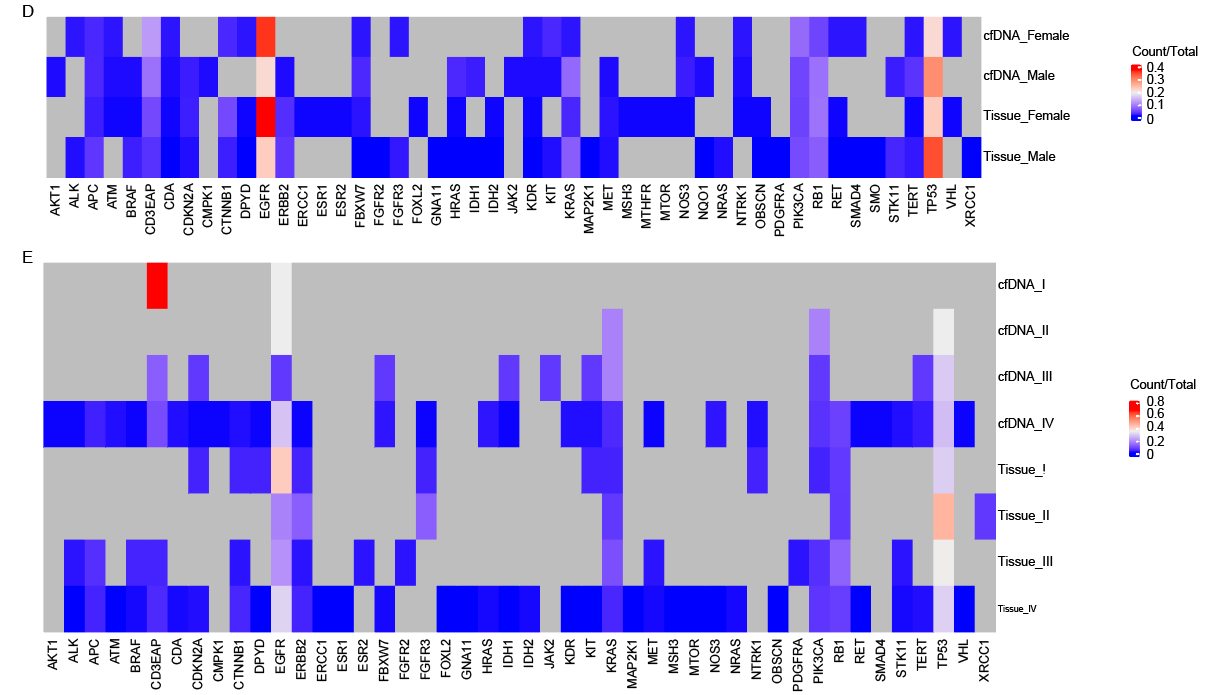


Supplementary **figure 1.** Concordance of genomic subtyping derived from tissue DNA or cfDNA in NSCLC patients with organ metastasis **(A)**, survival **(B)**, smoking **(C)**, gender **(D)**, and disease stage **(E)**.


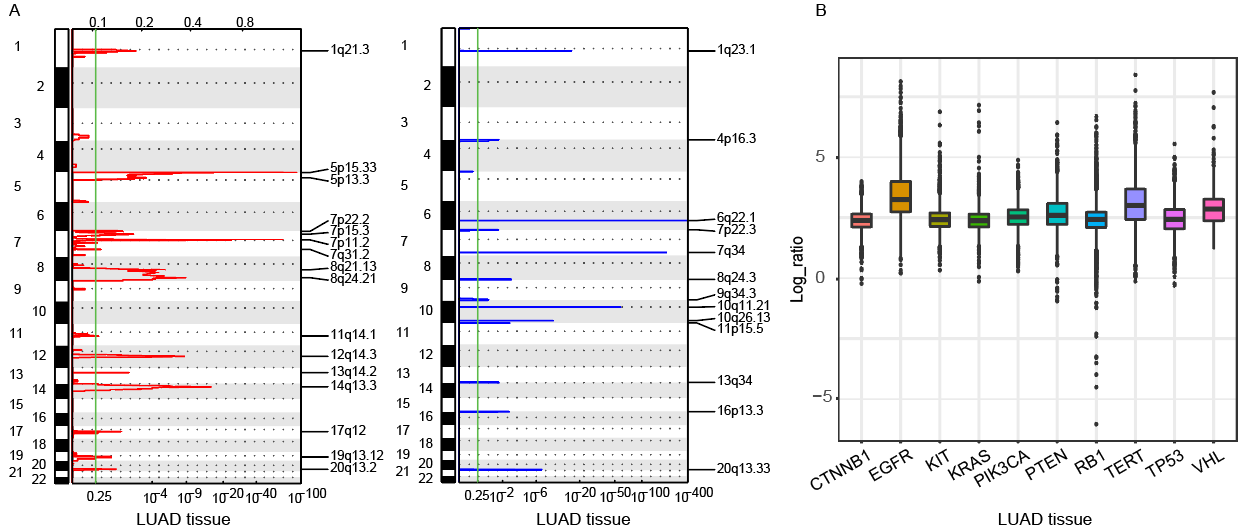

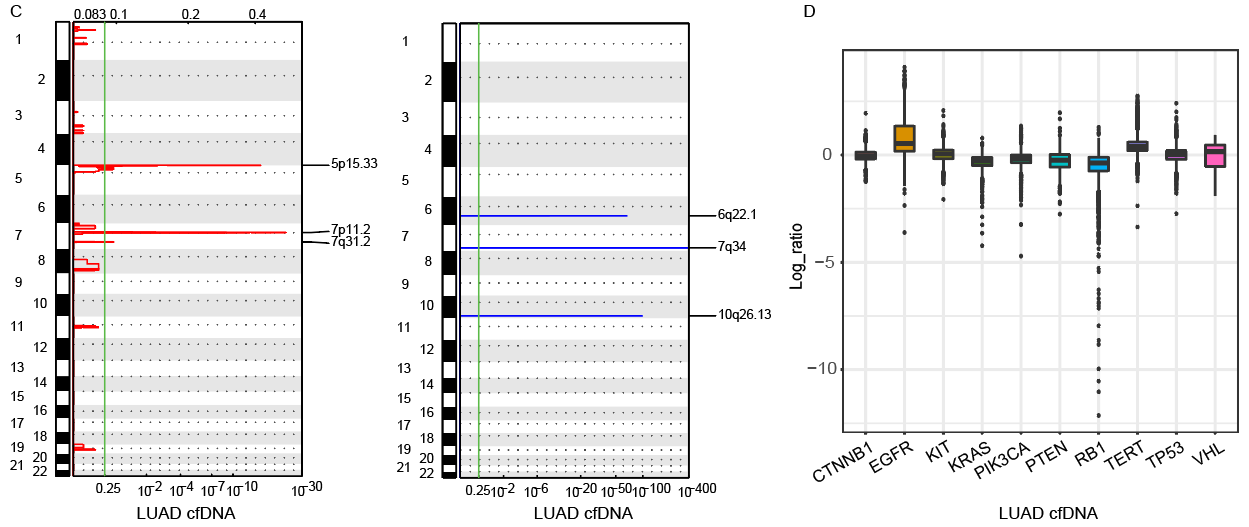


Supplementary **figure 2.** Somatic copy number alterations (SCNAs) of lung adenocarcinoma derived from NGS of tumor tissue samples (A, B) and cfDNA (C, D). (A) The significant peaks of copy number gain (left) and significant peaks of copy number loss (right) in lung adenocarcinoma tissue samples are plotted by chromosomal location (vertical axis) by CNVkit. (B) The 10 genes were examined by the hybridization capture-based NGS panel of 95 genes in lung adenocarcinoma tissue samples. (C) The significant peaks of copy number gain (left) and significant peaks of copy number loss (right) in lung adenocarcinoma plasma samples are plotted by chromosomal location (vertical axis) by CNVkit. (D) The 10 genes were examined by the hybridization capture-based NGS panel of 95 genes in lung adenocarcinoma plasma samples.


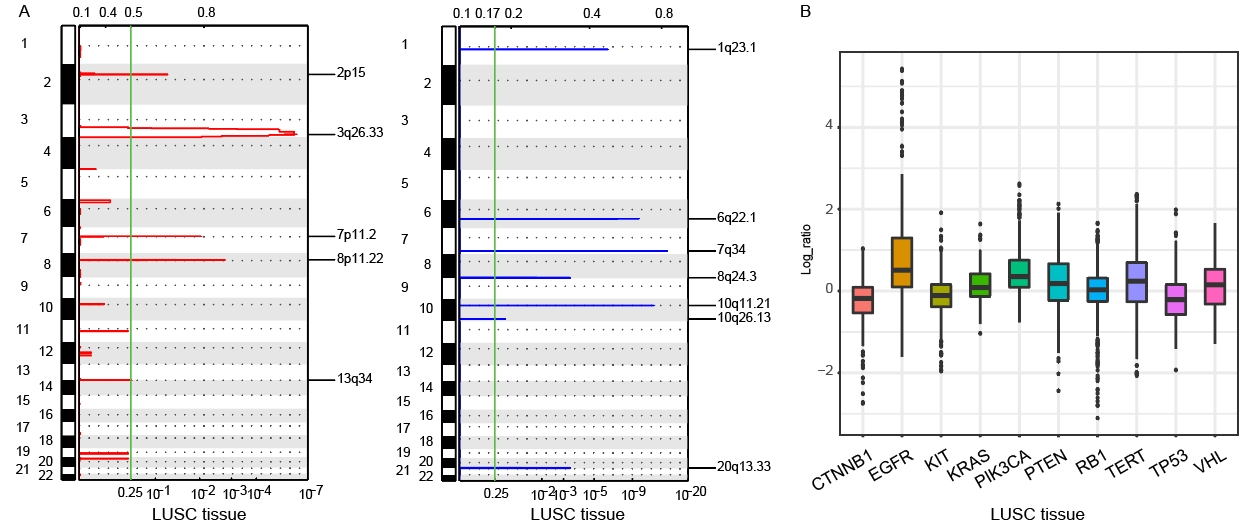

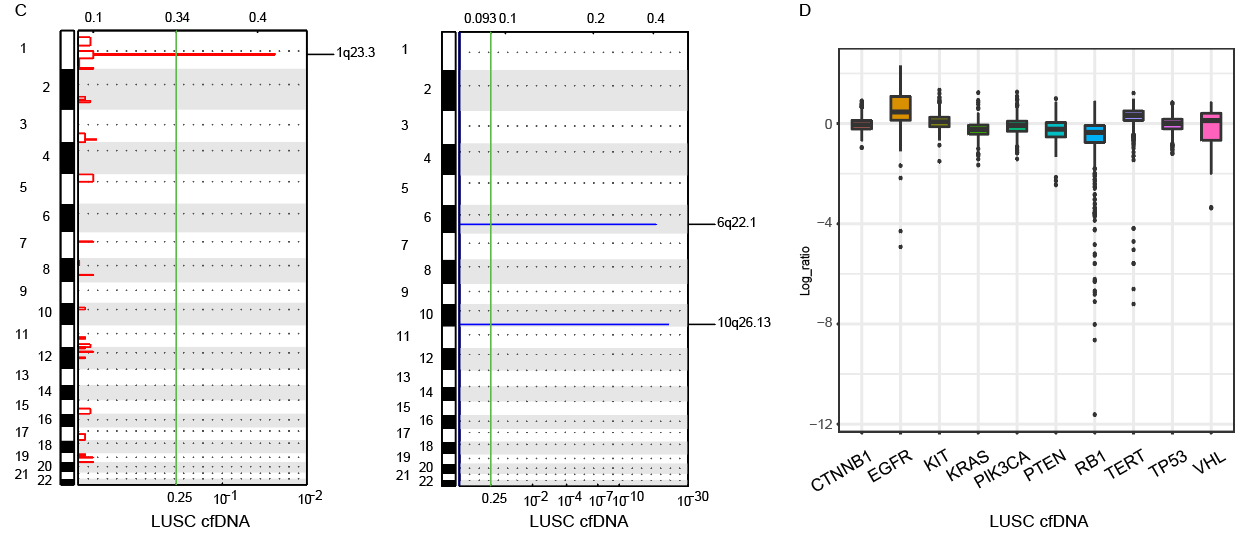


Supplementary **figure 3.** Somatic copy number alterations (SCNAs) of lung squamous carcinoma derived from NGS of tumor tissue samples (A, B) and cfDNA (C, D). (A) The significant peaks of copy number gain (left) and significant peaks of copy number loss (right) in lung squamous carcinoma tissue samples are plotted by chromosomal location (vertical axis) by CNVkit. (B) The 10 genes were examined by the hybridization capture-based NGS panel of 95 genes in lung squamous carcinoma tissue samples. (C) The significant peaks of copy number gain (left) and significant peaks of copy number loss (right) in squamous carcinoma plasma samples are plotted by chromosomal location (vertical axis) by CNVkit. (D) The 10 genes were examined by the hybridization capture-based NGS panel of 95 genes in lung squamous carcinoma plasma samples.


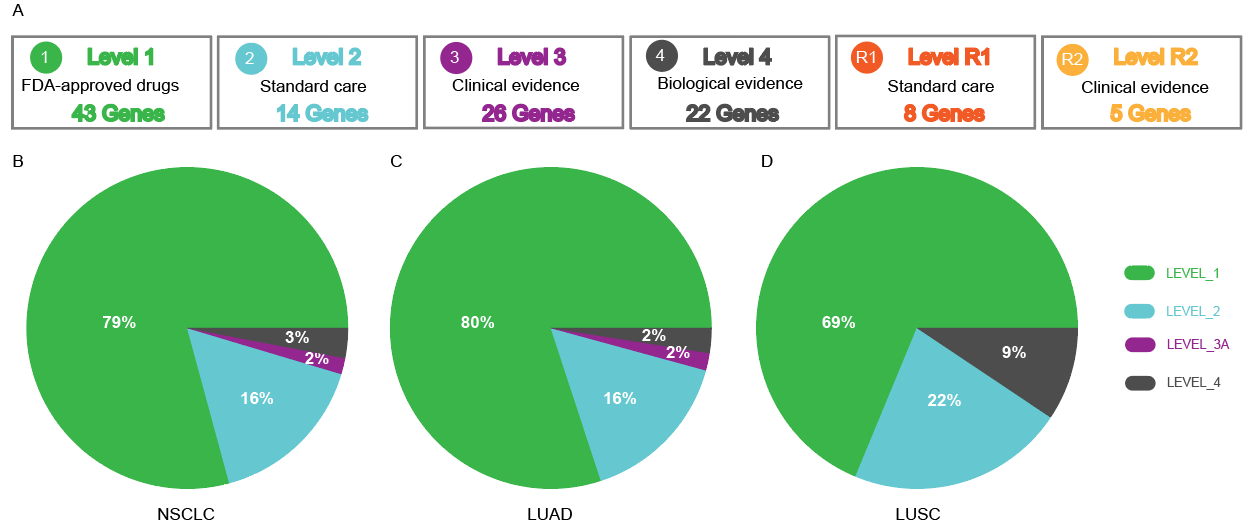

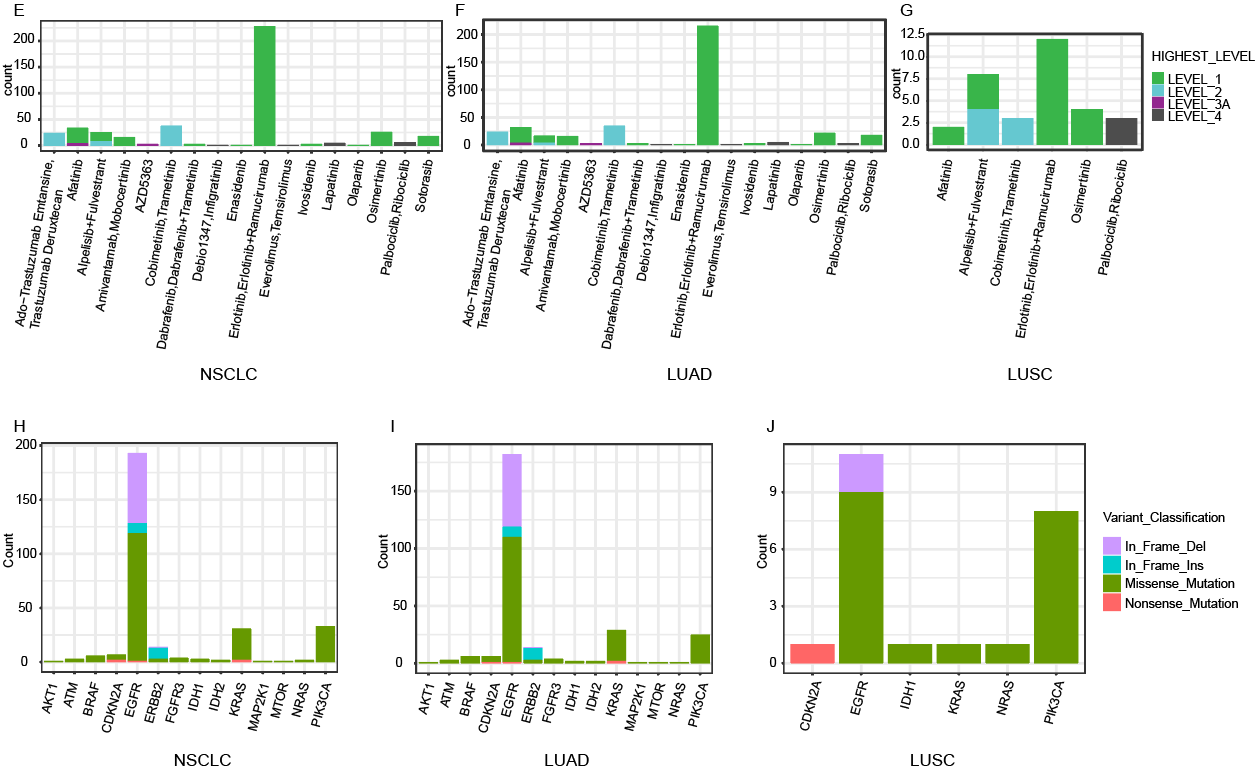


Supplementary **figure 4.** Clinical action ability of somatic mutations revealed by the hybridization capture-based NGS panel of 95 genes. Somatic mutations were defined based on their clinical evidence in terms of OncoKB (A). Samples were matched to the highest level of actionable alterations in NSCLC (n=395) (B)**,** LUAD (n=340) (C), and LUSC (n=54) (D). Distribution of actionable alterations in NSCLC (n=395) (E)**,** LUAD (n=340) (F), and LUSC (n=54) (G). Distribution of alteration types in NSCLC (H)**,** LUAD (I), and LUSC (J).
